# The “self” in pain: high levels of schema-enmeshment worsen fibromyalgia impact

**DOI:** 10.1186/s12891-021-04740-5

**Published:** 2021-10-12

**Authors:** Myrella Paschali, Asimina Lazaridou, Eric S. Vilsmark, Jeungchan Lee, Michael Berry, Arvina Grahl, Alessandra Anzolin, Marco Loggia, Vitaly Napadow, Robert R. Edwards

**Affiliations:** 1 Department of Anesthesiology, Harvard Medical School, Brigham & Women’s Hospital, 850 Boylston St, Suite 302, Chestnut Hill, MA 02467 USA; 2 Athinoula A. Martinos Center for Biomedical Imaging, Massachusetts General Hospital, Harvard Medical School, Charlestown, MA USA

**Keywords:** Chronic pain, Enmeshment, Fibromyalgia, Self, Schema-enmeshment, PRISM, Self-illness-separation

## Abstract

**Objective:**

Chronic pain can have detrimental effects on quality of life and a profound impact on one’s identity. The Pictorial Representation of Illness- and Self-Measure (PRISM), is a visual tool designed to measure the self-illness separation (SIS) that represents the degree of schema-enmeshment (i.e., the degree to which the self-schema and the illness-schema come to overlap). Our aim was to investigate the relationship between schema-enmeshment and pain-related outcomes in patients with fibromyalgia.

**Methods:**

In this cross-sectional study, 114 patients with fibromyalgia completed self-report assessments of pain catastrophizing, pain severity and interference, impact of symptoms, anxiety, and depression. SIS was assessed using an iPad version of PRISM. Mediation analyses evaluated the mediating role of schema-enmeshment on the association between pain catastrophizing and fibromyalgia impact.

**Results:**

A higher degree of schema-enmeshment was associated with greater pain catastrophizing, pain severity and interference, impact of symptoms, and depression. Moreover, a mediation analysis revealed that schema-enmeshment significantly mediated the association between pain catastrophizing and fibromyalgia impact (*p* < 0.001).

**Conclusions:**

Our results indicate that schema-enmeshment is associated with greater intrusiveness of chronic pain on everyday life, thereby posing significant limitations on the emotional and physical well-being of fibromyalgia patients. Schema-enmeshment also appears to partly account for the deleterious effect of pain catastrophizing on disease impact. The PRISM is a simple tool that may uniquely capture the extent to which chronic pain and illness infiltrates and affects one’s self-concept.

## Introduction

Chronic pain can substantially interfere with quality of life and daily functioning [1, 2] and is strongly associated with depression, anxiety, fatigue, and suffering [3–6]. Due to its enormous negative impact on one’s life, chronic pain can adversely affect a person’s identity or self-schema [7]. This might manifest as a loss of the sense of self and diminution of the defining aspects of a life (e.g., reductions in the sense of oneself as an accomplished, productive, socially engaged human being) as a result of living with chronic pain. The experienced loss of identity, meaning and autonomy are often considered to be a core component of suffering, especially the suffering associated with chronic pain [8, 9]. The Schema Enmeshment Model of Pain was developed in 2001 by Pincus and Morley to characterize and further elucidate changes in self resulting from chronic pain. This model consists of multiple schemas: self, illness, and pain-related schemas that can become enmeshed if their elements overlap significantly. The degree of schema-enmeshment (i.e., the degree to which the self-schema and the illness-schema come to overlap, such that the one’s identity is defined by illness) is thought to partly determine the emotional adjustment to chronic pain [9].

Although pain is nearly ubiquitous, the individual pain experience and suffering are multifaceted and related to numerous psychosocial constructs and processes [10]. Determining how to measure the impact of chronic pain on the self can be challenging, as the notion of “self” is an abstract, subjective experience. Measurements which incorporate patient perspectives on the self can offer insight into these intangible factors that otherwise evade traditional approaches of objective pain measurement. To date, typical methods for assessing the impact of chronic pain on the self have relied on lengthy questionnaires that require adequate language competency to complete. In contrast, the *Pictorial Representation of Illness and Self-Measure* (PRISM) encapsulates a single multifactorial measurement with simple instructions in a short amount of time. The PRISM is a simple test created by Büchi and colleagues [11] to measure what in German is *Leidensdruck*, the burden of suffering due to illness. Burden of suffering is defined as “a state of severe distress associated with events that threaten the intactness of the person” [12]. This term not only encompasses physical aspects of illness, but also the illness’s psychological effects and the extent to which it impedes a patient’s daily life and relationships. The PRISM measures the self-illness-separation (SIS) [8, 11] which quantifies the schema-enmeshment between self and illness and is equivalent to the degree to which the illness defines, intrudes upon, or threatens the sense of self [9, 13].

To complete the PRISM, participants are instructed to arrange two discs on a flat surface, labeled “self” and “illness”, according to their own experience with illness. The task can be conducted through a number of media including patients using a pencil and paper [14] or digital tools such as a mouse to move the illness circle on screen [13] or a touch screen [15]. The validity of the PRISM has been demonstrated numerous times for an array of chronic disorders including dizziness [14], psoriasis [16], tinnitus [15], and chronic pain [17]. The proximity of the discs quantifies the SIS score; the closer the 2 discs are to overlapping, the greater the enmeshment of self with concepts of illness. The SIS scores for these patient populations generally correlate negatively with measures of severity of illness, depression, and other adverse health-related quality of life impacts that are measured by typical patient-reported outcomes, i.e., a shorter distance between the discs is associated with greater illness severity, psychosocial impact of illness, etc.

The current literature shows increasing attention to the relationship on the self and pain [18]... The measure of illness identity that is most used in pain is the Illness Perception Questionnaire (IPQ) that includes the subscale “identity” [19]. The IPQ correlates moderately with catastrophizing; however, it doesn’t correlate with the SIS measured by the PRISM [13]. Further, Sun et al. found that illness identity and perceptions (measured using the IPQ) do not fully explain the effects of catastrophizing on pain [20]. These previous reports indicate that the PRISM assesses a distinct construct compared to the IPQ and highlight the importance of studying the SIS in relation to catastrophizing. Additionally, to our knowledge, no other study has utilized PRISM to investigate the relationship between the self and pain in fibromyalgia patients. Thus, the present study seeks to expand the existing literature by investigating the relationships between schema-enmeshment and pain-related outcomes such as pain catastrophizing in this patient population. Fibromyalgia is a complex chronic pain condition characterized by widespread pain, fatigue, disturbed sleep, anxiety, depression, cognitive problems and impaired functioning [21]. Prior work has suggested that patients with fibromyalgia demonstrate significant schema-enmeshment when compared to a control group [22]. The PRISM task has been validated for measuring the SIS in a mixed population of chronic pain sufferers [17], but it has only rarely been studied as a contributor to adverse chronic pain-related outcomes. We hypothesized that the distance between self and illness discs (i.e., the SIS) would be negatively correlated with fibromyalgia pain severity, pain-related catastrophizing, and other measures of distress. Furthermore, we hypothesized that schema-enmeshment would mediate the established associations of cognitive and affective factors (e.g., pain catastrophizing) with the severity and impact of chronic fibromyalgia pain, as prior studies have suggested that the degree of schema-enmeshment could determine emotional adjustment to chronic pain [9].

## Methods

This baseline data collection was part of a larger neuroimaging clinical study performed at the A. A. Martinos Center for Biomedical Imaging in Boston, Massachusetts. Patients diagnosed with fibromyalgia according to American College of Rheumatology criteria, which require the presence of widespread pain as well as several somatic and cognitive symptoms [23] were screened (*n* = 216).

The inclusion criteria used to screen potential participants were: (1) 18–75 years old, (2) female, (3) Wolfe et al.’s [21] research criteria for fibromyalgia diagnosis for at least 1 year, (4) at least 4 out of 10 average baseline pain intensity, and pain reported for at least 50% of days, (5) fluent in English, and able to provide written informed consent. The exclusion criteria were: (1) comorbid acute pain conditions or comorbid chronic pain conditions rated by the subject as more painful than fibromyalgia, (2) current use of stimulants (e.g. modafinil), (3) pregnant or nursing women, (4) psychiatric disorder with a history of psychosis (e.g. schizophrenia), (5) recent psychiatric hospitalization (past 6 months), (6) current or recent use of recreational drugs, (7) active suicidal ideation, (8) autoimmune or inflammatory disease (RA, SLE, IBD) that causes pain, (9) documented peripheral neuropathy of known cause, and (10) lower limb vascular surgery or current lower limb vascular dysfunction.

### Sociodemographic data

The collected sociodemographic information included age, marital status, race, current occupational status, and educational level..

### Clinical measures

#### Pain Catastrophizing

The Pain Catastrophizing Scale (PCS) is a 13-item, widely used, self-report measure of catastrophic thinking associated with pain. The PCS consists of three subscales: rumination, magnification, and helplessness. The PCS is well-validated in chronic pain and has shown good psychometric properties in pain patients and controls [24].

#### Pain severity and interference

To measure pain, we used the Brief Pain Inventory (BPI), a 15-item measure, that consists of two multi-item sub-scales that measure pain intensity and pain interference with daily activities. The BPI is well-validated in chronic pain populations and is frequently recommended as an outcome measure of pain severity and pain interference [25].

#### Fibromyalgia impact

The Fibromyalgia Impact Questionnaire-Revised (FIQR) is 21-question measure with an 11-point numeric rating scale (NRS) of 0 to 10, with 10 being “worst”. The questionnaire is divided into three domains assessing: (a) Function, (b) Overall impact, and (c) Fibromyalgia symptoms. All FIQR items reference a time frame of the past 7 days. The FIQR has sound psychometric properties [26].

#### Emotional distress

*Anxiety and Depression.* Participants completed the Patient-Reported Outcomes Measurement Information System (PROMIS) anxiety and depression short forms, which have been repeatedly validated in chronic pain populations [27, 28] including fibromyalgia [29]. The anxiety subscale consists of 7 items and measures the frequency with which emotions such as fear, stress, and anxiety are experienced by the patient on a scale from “never” to “always”. The depression subscale consists of 8 items that similarly measure the frequency with which emotions such as worthlessness, hopelessness, and sadness are experienced. Higher scores signify higher levels of emotional distress.

#### PRISM task

**Fig. 1:**
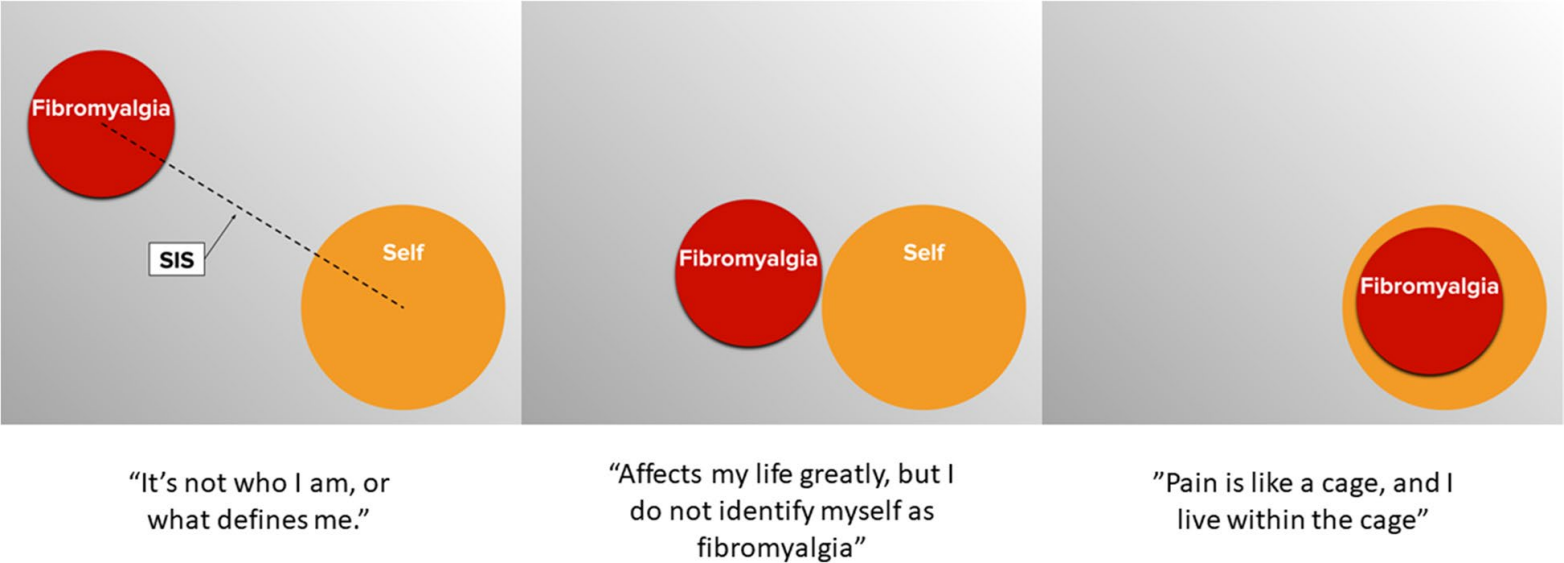
Examples of the Pictorial Representation of Illness and Self Measure (PRISM) task, with the red “illness” disc (fibromyalgia) and the yellow “self” disc)

We used an electronic version of the PRISM task on an iPad, which has been used in previous studies to assess the SIS [15, 30]. In order to permit a comparison with previous studies conducted using a white letter-sized metal board (DIN-A4) with a fixed, yellow circle (diameter 70 mm) glued in the bottom left-hand corner, all formats were normalized from the display of the iPad (147 × 196 mm) to DIN-A4 format (210 × 297 mm). The following descriptions are based on the DIN-A4 format. Participants were presented with a white panel taking up the whole display of the iPad, with a yellow “self” disc affixed to it at the bottom right-hand corner (diameter 70 mm) and a movable red “fibromyalgia” disc (diameter 50 mm) in the middle of the screen. Patients were asked to imagine that the white surface is their life at the present moment, the yellow fixed disc represents themselves and the colored red disc reflects their fibromyalgia illness. Patients were then instructed to place the red disc representing fibromyalgia, in relation to the fixed disc representing their self (Fig. 1). The PRISM was scored by measuring the distance from the center of the yellow “self” disc to the center of the red “fibromyalgia” disc. The distance in millimeters between the two discs is defined as the SIS (SIS range 0–256 mm) [11].

### Procedures

The data, including informed consent, completion of the above-mentioned questionnaires, and the PRISM task, was collected during a baseline visit of a larger neuroimaging study.

### Statistical analysis

Demographic and clinical characteristics were assessed and reported as means and standard deviations (SD) for continuous variables and percentages for dichotomous variables. Similar to two previous studies in orofacial pain and tinnitus patients [15, 31], SIS was divided into two groups reflecting the degree of schema-enmeshment. In the low-SIS group, the red “fibromyalgia” disc was overlapping with or was completely placed inside the yellow self-circle, i.e., patients were placed in this group if they reported a SIS smaller than 60 mm (overlap is represented by smaller values than the sum of both circle radii: self 35 mm + illness 25 mm). In the high-SIS group the red “fibromyalgia” disc was placed outside the yellow “self” disc (SIS ≥60 mm). Possible differences between participants with low and high SIS scores were assessed utilizing a Mann-Whitney test for independent samples separately on each clinical measure and a chi-square test for sociodemographic measures. In addition, we calculated Pearson correlations to explore associations between all variables. We then performed hierarchical linear regression analyses predicting fibromyalgia impact (FIQR). Finally, we performed mediation analysis (PROCESS, model 4) to examine whether the SIS mediated the relationship between pain catastrophizing (PCS) and fibromyalgia impact (FIQR). A custom written macro (PROCESS; www.processmacro.org) for SPSS (v26, IBM, USA) was used to perform multiple mediation pathway analysis with bias-corrected bootstrapping tests. Bootstrapping is a statistical method that involves drawing repeated samples from the data with replacement to gain multiple estimates (5000 bootstrap samples) of the indirect effect attributed to potential mediator variables [32, 33]. Bootstrapping provides a way of circumventing power deficiencies of normal theory tests (i.e. Sobel) typically introduced by the non-normality in the sampling distribution [34, 35]. Bias-corrected 95% confidence intervals were produced for each potential mediator and were used to test the significance of the total and indirect (i.e. mediation) effects. Estimates of indirect effects were considered significant in the case that zero was not included within the confidence intervals [36].

## Results

### Subject characteristics

One-hundred-and-fourteen (*N* = 114) participants with fibromyalgia were eligible for participation. Within this group, 92 provided complete data regarding the PRISM task. The average age of patients was 41.2 ± 12.4 years. The mean SIS was 62 mm and the median was 36 mm, indicating that the majority of participants placed the “fibromyalgia” disc in such a way that the “self” and “fibromyalgia” discs were overlapping. Further sociodemographic and clinical characteristics are reported in Table 1.

**Table 1:** Sociodemographic and clinical variables

Sociodemographic variables		*n* = 114
Age (mean ± SD)		41.2 ± 12.4
Caucasian		76%
Employed		54.%
Married		36.%
Living alone		16.50%
Education Level (college degree)		60%
Annual Income (above $45000)		59.1%
Clinical variables	Scale Score Range	Mean ± SD
SIS (*n* = 92)	0-256 mm	62 ± 82 mm
PCS (n = 114)	0–52	23.7 ± 12.2
BPI (Severity) (n = 112)	0–10	5.1 ± 1.8
BPI (Interference) (*n* = 112)	0–10	5.7 ± 2.4
FIQR (n = 112)	0–100	56.4 ± 16.8
Anxiety (PROMIS) (n = 110)	40.3–81.6	59.0 ± 8.0
Depression (PROMIS) (*n* = 110)	37.1–81.1	57.8 ± 9.0

Note. *SD* standard deviation; *SIS* Self-Illness-Separation; *BPI* Brief Pain Inventory; *PCS* Pain Catastrophizing Scale; *FIQR* Fibromyalgia Impact Questionnaire-Revised; *PROMIS* Patient-Reported Outcomes Measurement Information System (T-Score)

### Associations between the SIS and demographic and clinical characteristics

No significant results were found correlating the SIS score with age, employment, marital status, level of education and income. SIS was negatively correlated with pain catastrophizing, pain severity and interference, fibromyalgia impact, and depression. SIS did not correlate with anxiety (Table 2).

**Table 2:** Correlations with Self-Illness-Separation (SIS)

	SIS	PCS	BPI Sev.	BPI Interf.	FIQR	Anxiety	Depression
SIS	–	−.37^b^	−.28^b^	−.24^a^	−.37^b^	−.09	−.28^b^
PCS	−.37^b^	–	.50^b^	.59^b^	.59^b^	.52^b^	.58^b^
BPI Severity	−.28^b^	.50^b^	–	.73^b^	.67^b^	.22^a^	.31^b^
BPI Interference	−.24^a^	.59^b^	.73^b^	–	.78^b^	.37^b^	.51^b^
FIQR	−.37^b^	.59^b^	.67^b^	.77^b^	–	.42^b^	.53^b^
Anxiety (PROMIS)	−.09	.52^b^	.22^a^	.37^b^	.42^b^	–	.60^b^
Depression (PROMIS)	−.28^b^	.58^b^	.31^b^	.51^b^	.53^b^	.60^b^	–

*Note*. ^a^Correlation is significant at the 0.05 level (two-tailed); ^b^Correlation is significant at the 0.01 level (two-tailed); *PCS* Pain Catastrophizing; *BPI* Brief Pain Inventory; *FIQR* Fibromyalgia Impact Questionnaire-Revised; *PROMIS* Patient-Reported Outcomes Measurement Information System (T-Score)

### Clinical characteristics of participants with low and high SIS

The means of the clinical characteristics for each PRISM group and the results of their statistical comparison (Mann-Whitney test) can be found in Table 3. Patients reporting a higher degree of schema-enmeshment (low SIS group; mean SIS 20 mm) had higher scores for catastrophizing, pain severity and interference, fibromyalgia impact, anxiety, and depression compared to patients with lower schema-enmeshment (high SIS group; mean SIS 144 mm). There were no significant differences in the sociodemographic characteristics of the low and high SIS groups.

**Table 3:** Clinical and sociodemographic characteristics of participants with low and high SIS (mean ± SD)

	Low SIS (high enmeshment) (*n* = 61)	High SIS (low enmeshment) (*n* = 31)	*p*-value
Age	42.7 ± 12.33	38.65 ± 11.64	.203
Caucasian (n)	47	24	.326
Employed (n)	29	19	.212
Married (n)	20	11	.796
Living alone (n)	11	3	.292
Education Level (college degree) (n)	38	21	.607
Annual Income (above $45000) (n)	35	22	.204
PCS	25.66 ± 11.60	17.61 ± 10.78	.002
BPI Severity	5.59 ± 1.62	4.27 ± 1.92	.001
BPI Interference	6.28 ± 2.11	4.56 ± 2.41	.001
FIQR	62.00 ± 13.39	46.36 ± 16.76	.000
Anxiety (PROMIS)	60.27 ± 7.74	56.40 ± 9.20	.045
Depression (PROMIS)	59.79 ± 8.83	53.81 ± 8.54	.002

Note. *SIS*Self-Illness-Separation; *PCS* Pain Catastrophizing; *BPI* Brief Pain Inventory; *FIQR* Fibromyalgia Impact Questionnaire-Revised; *PROMIS* Patient-Reported Outcomes Measurement Information System (T-Score).

### SIS as a predictor of disease impact

Results of a linear regression predicting the level of fibromyalgia impact (FIQR) showed that catastrophizing and the SIS collectively explained 32% of the variance in FIQR (*F* (2,87) = 20.88, *p* < 0.001, *r*^*2*^ = .324). The individual predictors were examined further and indicated that catastrophizing (*p* < 0.001) and the SIS (*p* < 0.05) were significant predictors for level of fibromyalgia impact (FIQR) in the model (Table 4).

**Table 4:** Linear Regression Analyses Results for Variables Predicting Fibromyalgia Impact (FIQR)

	*B*	*SE B*	*β*
Pain Catastrophizing (PCS)	.64	.13	.47***
Self-Illness-Separation (SIS)	−.58	.28	−.19*

*Note*. *r*^2^ = .32, F = 20.88***; *p < .05. **p < .01. *** *p* < .001

### SIS as a mediator of the relationship between catastrophizing and fibromyalgia impact

**Fig. 2:**
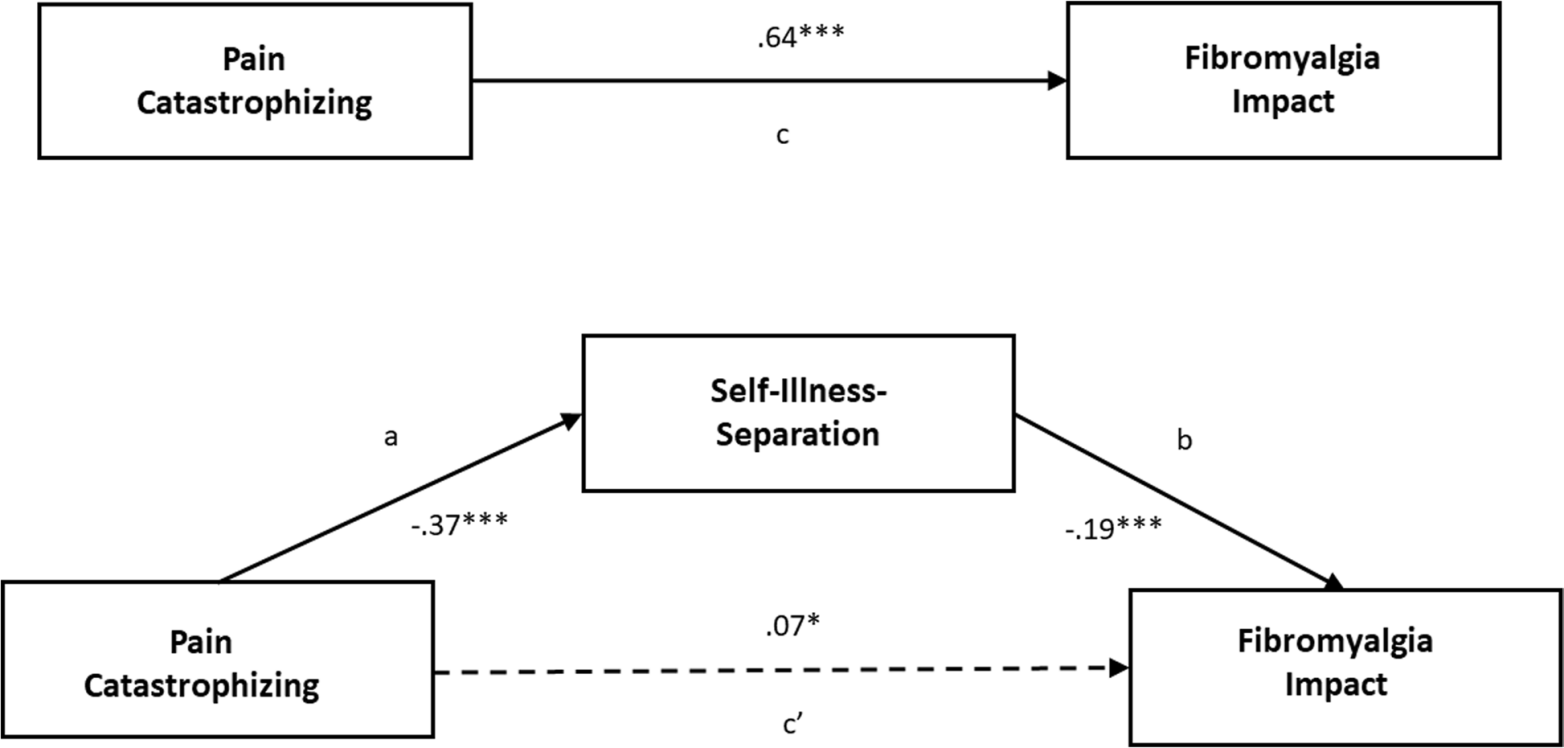
Self-Illness-Separation mediating the relationship between pain catastrophizing and fibromyalgia impact. * significant at p ≤ 0.05; ** significant at *p* ≤ 0.01, *** significant at p ≤ 0.001

Figure 2 depicts the results of the mediation analysis. The results indicate that SIS mediated the influence of pain catastrophizing on fibromyalgia impact. All paths within the model were significant: pain catastrophizing was negatively associated with SIS (path a = −.37, *p* < .001) and positively associated with fibromyalgia impact (path c = .64, *p* = < .001). SIS was significantly negatively associated with fibromyalgia impact (path b = −.19, *p* < .001).

## Discussion

The present study aimed to assess the interplay of psychosocial factors with patients’ perceived degree of schema-enmeshment measured using the PRISM task in patients with fibromyalgia. Our results support the hypothesized relationship between schema-enmeshment with pain catastrophizing, clinical pain severity, fibromyalgia impact, and depression. The closer the patients placed the ‘illness’ disc relative to the ‘self’ disc (greater schema-enmeshment), the more intense daily fibromyalgia pain they experienced and the more catastrophizing, depression, and fibromyalgia impact on their lives they reported. These findings are broadly consistent with previous validation studies of the PRISM [8, 17]. Our results also indicated that greater schema-enmeshment was associated with greater intrusiveness of the illness into patients’ everyday life. Our results also suggested that the more schema-enmeshment patients are reporting the more significant the fibromyalgia-related limitations are in their emotional and physical well-being. A similar pattern of results was obtained in a different population with a chronic illness [16]. An important observation was that sociodemographic variables did not correlate with the degree of schema-enmeshment, which suggests that our results may be applicable to participants of different age and socioeconomic groups. It is interesting to note that the average SIS of fibromyalgia patients in our study (mean = 62 mm) was substantially lower compared to other chronic diseases such as tinnitus (mean = 93 mm; iPad version, results normalized to a DIN-A4 format.) [15], or lupus erythematosus (mean = 140 mm; paper version - DIN-A4 format) [37]. While the present study does not include a chronic pain comparison group, it is possible that the complex and disabling nature of fibromyalgia is characterized by greater schema-enmeshment and suffering than other chronic diseases in which the PRISM has been used.

Both pain catastrophizing and schema-enmeshment were found to be significant predictors for fibromyalgia impact, suggesting that fibromyalgia patients high in catastrophizing and schema-enmeshment are more likely to experience a higher fibromyalgia impact. This aligns with previous findings [38] on the association of pain catastrophizing and illness identity. Prior reviews have noted that pain catastrophizing is common in individuals with fibromyalgia [39] and has been shown to be the most consistent psychosocial factor predicting adjustment to chronic pain and contributing to a delayed recovery from chronic musculoskeletal pain [40]. Results of a previous study showed that patients with fibromyalgia who endorsed greater levels of catastrophizing perceived their disease impact to be higher compared to patients with lower catastrophizing scores [41]. We therefore examined the role schema-enmeshment plays in the relationship between pain catastrophizing and fibromyalgia impact. Our mediation analysis results indicate that the degree of schema-enmeshment serves as a mediator between catastrophizing and fibromyalgia impact, indicating that increased schema-enmeshment acts as one potential pathway by which catastrophizing exerts a deleterious effect on disease impact.

Broadly, the present findings highlight the importance of illness beliefs in shaping pain-related outcomes in fibromyalgia and illuminate the potential benefits of incorporating measures of identity and self-concept, such as the PRISM, in biopsychosocial studies of chronic pain. Taken together, our results indicate that schema-enmeshment might be one of the pathways implicated in the maintenance of interfering fibromyalgia symptoms in the patient’s life. Such results may have important clinical implications for clinicians involved in the treatment of patients with fibromyalgia. The use of PRISM in clinical settings can efficiently help to identify patients with a greater burden of suffering and with greater identity vulnerability. The acquired information could then be utilized in the determination of the most suitable treatment option for the patient. For example, a patient with high schema-enmeshment could be a good candidate for Acceptance and Commitment Therapy (ACT), since schema-enmeshment is related to one’s adjustment to or acceptance of their chronic illness [42]. Additional benefits of using the PRISM in clinical settings are its simple and fast administration and the facilitation of an in-depth discussion about the impact of fibromyalgia has on the patient’s identity during, for example, a psychotherapeutic session.

Our study has some limitations which are worth noting. Firstly, the pain-related outcomes were measured only using self-report measures, which can be subject to a variety of biases. Secondly, although this study has identified associations between schema-enmeshment and pain-related variables, its cross-sectional nature does not allow conclusions as to cause and effect. That is, whether high levels of pain catastrophizing, depression and disease severity cause elevations in enmeshment over time, or whether the enmeshment of self and illness contributes causally to pain impact, catastrophizing, and other adverse outcomes is unclear. Longitudinal studies will be necessary to definitively answer these questions. It is known that psychosocial factors can interact with pain experiences in a non-linear and complex way; psychosocial characteristics can serve as vulnerability factors [43] or develop for the first time as a response to the ongoing pain experience [44]. We suggest that the relationship between schema-enmeshment and the investigated pain-related variables is likely to be bi-directional, but prospective research is needed to shed light on the temporal dynamics of these relationships. Lastly, our study did not utilize a conventional measure for illness identity such as the IPQ.

## Conclusions

The present study uses a novel diagnostic instrument, a fairly large clinical sample, and formal mediation analysis to explore the role of schema-enmeshment among patients with fibromyalgia. Our results illustrate that the PRISM can serve as a unique tool capturing the extent to which chronic pain and illness infiltrates and affects one’s self-concept. Taking into consideration that PRISM is easy and fast to administer, we recommend that PRISM might serve as an additional valuable diagnostic tool in clinical contexts assessing the burden of suffering and schema-enmeshment in patients with fibromyalgia. Although not investigated in the present study, we expect that a reduction of schema-enmeshment could potentially lead to improvement of pain-related outcomes. We propose that nonpharmacologic, psychosocially-oriented treatments such as cognitive behavioral therapy and ACT (which have demonstrated efficacy in FM [45, 46]) may be suitable interventions in this regard. The effectiveness of interventions aiming to reduce the degree of schema-enmeshment, and the mechanisms by which those interventions affect pain-related outcomes, should be explored in future studies.

## Data Availability

The datasets used during the current study are available from the corresponding author on reasonable request.

## Abbreviations

PRISM: Pictorial Representation of Illness- and Self-Measure
SIS: Self-Illness Separation
PCS: Pain Catastrophizing Scale
BPI: Brief Pain Inventory
FIQR: Fibromyalgia Impact Questionnaire-Revised
PROMIS: Patient-Reported Outcomes Measurement Information System
SD: Standard Deviation
ACT: Acceptance and Commitment Therapy
